# Vitamin D and assisted reproduction technologies: current concepts

**DOI:** 10.1186/1477-7827-12-47

**Published:** 2014-05-31

**Authors:** Valeria S Vanni, Paola Vigano', Edgardo Somigliana, Enrico Papaleo, Alessio Paffoni, Luca Pagliardini, Massimo Candiani

**Affiliations:** 1 Department Obstetrics and Gynecology, San Raffaele Scientific Institute, Via Olgettina 60, 20136 Milano, Italy; 2 Department Obstetrics and Gynecology, Fondazione Cà Granda, Ospedale Maggiore Policlinico, Via Commenda 12, 20122 Milano, Italy; 3 Università Vita-Salute, San Raffaele Scientific Institute, Via Olgettina 58, 20132 Milano, Italy

**Keywords:** Vitamin D, 25-hydroxyvitamin D, Controlled ovarian hyperstimulation (COH), Assisted reproduction technology (ART), In vitro fertilization (IVF), Infertility, Meta-analysis

## Abstract

Accumulating evidence from animal and human studies suggests that vitamin D is involved in many functions of the human reproductive system in both genders, but no comprehensive analysis of the potential relationship between vitamin D status and Assisted Reproduction Technologies (ART) outcomes is currently available. On this basis, the purpose of this systematic review and meta-analysis was to perform an in-depth evaluation of clinical studies assessing whether vitamin D status of patients undergoing ART could be related to cycle outcome variables. This issue is of interest considering that vitamin D deficiency is easily amenable to correction and oral vitamin D supplementation is cheap and without significant side effects. Surprisingly, no studies are currently available assessing vitamin D status among male partners of couples undergoing ART, while seven studies on vitamin D status of women undergoing controlled ovarian hyperstimulation (COH) for ART were found and included in the review. Results show that vitamin D deficiency is highly prevalent among women undergoing COH, ranging from 21% to 31% across studies conducted in Western countries and reaching 75-99% in Iranian studies. Data on vitamin D deficiency (25-hydroxyvitamin D serum levels <20 ng/ml) in relation to ART outcomes could be extracted from three studies and included in the meta-analysis, yielding a common risk ratio (RR) of 0.89 (95% CI 0.53-1.49) and showing a lower but not statistically significant likelihood of clinical pregnancy for vitamin-D-deficient women compared with vitamin-D-sufficient patients. In conclusion, there is insufficient evidence to support the routine assessment of vitamin D status to predict the clinical pregnancy rate in couples undergoing ART. The partly conflicting results of the available studies, potentially explaining the lack of statistical significance for a negative influence of vitamin D deficiency on clinical pregnancy rate, are likely secondary to confounders and insufficient sample size, and further larger cohort and randomised controlled studies are required.

## Background

Accumulating evidence from animal and human studies suggests that vitamin D is involved in many functions of the human reproductive system in both genders. While crucial roles for vitamin D in human infertility, pregnancy and neonatal growth have been extensively reviewed elsewhere [1-5] no comprehensive analysis of the potential relationship between vitamin D and Assisted Reproduction Technologies (ART) outcomes is currently available. This is surprising considering that there is a strong rationale for a potential role of vitamin D [4].

### Vitamin D and female reproductive function

A role for vitamin D in ovarian steroidogenesis is well established [4], and recent evidence has shown an effect of vitamin D on uterine receptivity [4,6]. Importantly, vitamin D has also been shown to be involved in the pathophysiology of some disorders of women of childbearing age that are most commonly encountered among women undergoing In Vitro Fertilization (IVF) procedures:

1. Vitamin D deficiency has been suggested to contribute to the development of insulin resistance and impaired glucose clearance in Polycystic Ovary Syndrome (PCOS). Indeed, even if the issue of whether PCOS itself may be associated with altered levels of vitamin D is still controversial, lower 25-hydroxyvitamin D [25(OH)D] levels characterise PCOS women suffering from metabolic disturbances including obesity, metabolic syndrome and insulin resistance [7,8]. Consistently, especially among PCOS patients with severe insulin resistance, a therapeutic efficacy of supplementation with vitamin D in improving insulin sensitivity, androgen levels, ovarian folliculogenesis and menstrual frequency was reported [9-11]. Surprisingly, however, no data regarding a potential role for vitamin D status in predicting or ameliorating IVF outcome among women with PCOS and metabolic disturbances are currently available.

2. Vitamin D status has recently been related to the development of uterine leiomyomas, with observations showing that lower 25(OH)D levels correlate with a higher risk and a greater volume of uterine fibroids, both in black and white ethnicities [12,13]. These findings were also confirmed in our study conducted in infertile women [14]. A potential therapeutic benefit of vitamin D supplementation in the inhibition of development and/or growth of uterine fibroids has been consistently established both *in vitro*[15-18] and in animal studies [19]. However, no evidence is currently available addressing the benefits of treatment with vitamin D for women with leiomyomas, neither in the context of IVF studies or in the general gynaecological population.

3. Endometriosis has also been associated with altered levels of 25(OH)D. In a preliminary case–control study by our group [20], we observed higher serum levels in affected cases but our results were subsequently properly questioned because we included in the control group a high proportion of women with leiomyomas and unexplained infertility, two conditions that were subsequently demonstrated to be deficient for 25(OH)D. More robust evidence recently emerged, showing that women within the highest quintile of predicted vitamin D concentration had a 24% lower risk of endometriosis than women in the lowest quintile [21]. Consistently, two animal studies have shown that treatment with vitamin D or with a Vitamin D Receptor (VDR) agonist can inhibit the development of endometriotic lesions [22,23], but the hypothesis of a beneficial effect of vitamin D supplementation in the treatment of patients with endometriosis has not yet been clinically tested.

4. Several lines of evidence show an association between vitamin D deficiency and Body Mass Index (BMI). Indeed, according to a recent meta-analysis totalling over 42,000 general adult patients, each 10% increase in BMI leads to a 4% decrease in 25(OH)D concentrations [24]. Vitamin D deficiency could therefore contribute to the adverse health effects associated with obesity [24], including lower clinical and live birth rates and higher miscarriage rate following ART [25]. However, no studies have so far considered the possibility of treating vitamin D deficiency in overweight women undergoing ART as a means of alleviating the adverse influences of raised BMI on ART outcomes.

### Vitamin D and male reproductive function

Vitamin D may play a role in human spermatogenesis. The favourable effect of 25(OH) on human spermatozoa has been shown *in vitro*[26-28] and three cross-sectional association studies on serum 25(OH)D levels and semen quality have been conducted in young healthy men without infertility problems [27,29,30]. The results, however, were quite discordant (Table 1). A single cross-sectional study [31] has also investigated the relationship between vitamin D status and semen parameters in a population of n = 364 infertile men as compared to n = 195 age-matched fertile controls. Interestingly, after adjustment for some relevant confounders, statistically significant associations were observed between 25(OH)D levels and sperm motility (Spearman’s coefficient = 0.12, p = 0.03) and morphology (Spearman’s coefficient = 0.12, p = 0.03) in infertile men (Table 1).

**Table 1:** Studies investigating the relationship between vitamin D status and semen parameters in healthy and infertile men

**Author, year, [reference #]**	**Country**	**Study design**	**Cases included**	**Sample size**	**Groups of patients identified by serum 25(OH)D levels**	**Statistical analysis used**	**Main finding(s)**	**Main confounding factors considered**
Ramlau-Hansen *et al.*, 2011 [29]	USA	Cross-sectional	Healthy men aged 18-21	307	3-24 ng/ml: low (33.5%) 25–37 ng/ml: medium (33.5%) 38–91: high (33%)	Spearman’s rank correlation test; Multivariable linear regression	No significant association between 25(OH)D levels and sperm parameters	Season
								BMI
								History of diseases
								Duration of abstinence
								Time from ejaculation to analysis
Blomberg Jensen *et al*., 2011 [27]	USA	Cross-sectional	Healthy men aged 18-21	300	<10 ng/ml: deficient (12%) 10-20 ng/ml: insufficient (32.7%) 21-30 ng/ml : sufficient (41.7%) >30 ng/ml: high (13.7%)	Kruskal Wallis test; Multivariable linear regression	Positive association between 25(OH)D levels and sperm progressive motility and morphology	FSH
								Duration of abstinence
								Time from ejaculation to analysis
								Serum calcium levels
								Season
Hammoud *et al*., 2012 [30]	USA	Cross-sectional	Healthy men aged 18-67	147	<20 ng/ml: deficient (12.4%) 20-49 ng/ml: intermediate (75.2%) ≥50 ng/ml: high (12.4%)	Multivariable linear regression	Negative association of both deficient and high 25(OH)D levels with sperm parameters	Season
								Age
								BMI
								Alcohol intake
								Smoking
Yang et al., 2012 [31]	China	Cross-sectional	Healthy and infertile men aged 20-40	195 (healthy group); 364 (infertile group)	<10 ng/ml: severely deficient 10-20 ng/ml: deficient 21-30 ng/ml: insufficient >30 ng/ml : sufficient	Spearman’s rank correlation test; Multivariable linear regression	Positive association between 25(OH)D levels, sperm motility and morphology also in the infertile group	Testosterone
								Season
								Duration of abstinence
								Time from ejaculation to analysis

Based on these observations, the purpose of this systematic review and meta-analysis was to perform an in-depth evaluation of clinical studies assessing whether vitamin D status of patients undergoing ART could be related to cycle outcome variables. This issue is of particular interest considering that vitamin D status may be easily adjustable.

## Review

### Methods

#### Study selection and search strategy

The present literature overview was conducted according to the Preferred Outcome Items for Systematic Reviews and Meta-analysis (PRISMA statement) [32]. Literature searches were conducted to identify studies published between January 1990 and February 2014. The electronic databases PUBMED, EMBASE and CENTRAL were searched using the following mixture of Medical Subject Heading (MeSH) terms and keywords terms: (‘Vitamin D’ OR ’25 (OH) D’ OR ‘1,25 (OH)2 D’ OR ‘calciferol’ OR ‘ergocalciferol’ OR ‘cholecalciferol’ OR ‘calcitriol’) AND (‘In Vitro Fertilization’ OR ‘Assisted Reproduction Technologies’). The search was not limited by language. Clinical trials assessing vitamin D status of patients undergoing ART and reporting any association between vitamin D status and IVF/Intracytoplasmatic Sperm Injection (ICSI) cycle outcome variables were considered eligible. The outcomes of interest for this systematic review included assessment of vitamin D levels throughout controlled ovarian hyperstimulation (COH), prevalence of vitamin D deficiency among patients undergoing ART, clinical pregnancy rate (CPR) and live birth rate (LBR). All pertinent reports were retrieved and the relative reference lists were systematically searched in order to identify any potential additional studies that could be included. Only those that were published as full-length articles were considered. Abstracts of scientific meetings were not included [33]. Eligibility assessment and data extraction were performed independently by two investigators (VSV and PV).

#### Data extraction

For each study, the following information was extracted: first author's last name; year of publication; country of origin; number of subjects; design of the study; statistical analysis; outcomes assessed; confounding factors considered; results. The only cycle outcome parameter assessed in all studies was CPR. Outcome data on CPR were therefore retrieved from individual reports and pooled estimation and meta-analysis were performed as follows. The number of patients who achieved a clinical pregnancy among groups of women with and without serum 25(OH)D deficiency (<20 ng/ml) was obtained from each study and used in the meta-analysis. A clinical pregnancy was defined as the observation of an intrauterine gestational sac following IVF/ICSI procedures and fresh embryo transfer. In one study [34], patients were divided into groups based on serum cut-off values that were different from 20 ng/ml (i.e. three groups based on serum vitamin D levels <10 ng/ml; 10–29 ng/ml and ≥ 30 ng/ml respectively). On the other hand, groups with <10 ng/ml and ≥30 ng/ml levels could be considered subgroups of patients with and without vitamin D deficiency respectively and were therefore included in the meta-analysis. Conversely, the intermediate group [serum 25(OH)D levels 10–29 ng/ml] could not be categorized neither as vitamin D deficient nor as vitamin D sufficient and was therefore not considered. Studies categorising patients only based on follicular fluid (FF) rather than serum levels of 25(OH)D were excluded from the meta-analysis in order to contain the heterogeneity.

#### Statistical analysis

The heterogeneity Cochrane Q and the I2 statistics were calculated, showing a moderate heterogeneity (I2 = 53%); for this reason the DerSimonian-Laird random-effects model, which incorporates both within- and between-study variations was used in the meta-analysis [35,36]. Due to the very low number of studies included in the meta-analysis, no statistical test for a small-study effect was conducted [37]. Results are expressed as risk ratio (RR) with 95% confidence intervals (CIs) [37]. Statistical significance was set at a p value of <0.05 and analysis was performed using Review Manager (RevMan, Version 5.2. Copenhagen: The Nordic Cochrane Centre, The Cochrane Collaboration, 2012).

## Results

**Figure 1:**
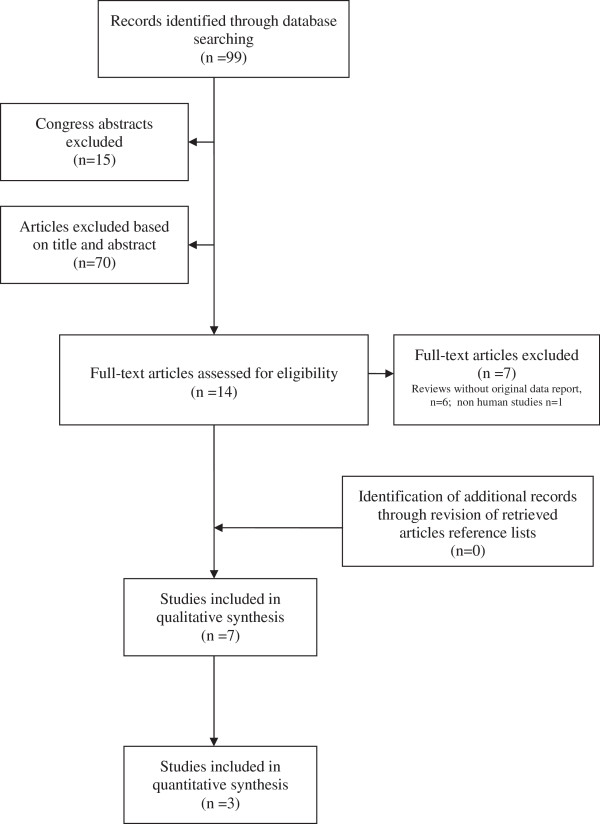
Flow diagram of the complete study selection process.

Figure 1 shows the flow diagram of the complete selection process following the PRISMA statement [32]. The database search initially identified 99 articles. Conference abstracts were excluded (n = 15). Of the 84 full-length articles, 14 potentially reporting on vitamin D status with regards to ART outcomes were retrieved for detailed assessment. After exclusion of seven full length articles for various reasons (*in vitro* studies n = 1, reviews without original data report, n = 6; Figure 1), a total of seven studies on the association between vitamin D status of ART patients and IVF/ICSI cycle outcome variables were identified. Their main methodological characteristics and findings are presented in Table 2. All of them were cohort studies. A total of three studies were conducted in North America, two in Iran, one in Greece and one in Israel.

**Table 2:** Studies investigating the relationship between vitamin D status and outcomes in women undergoing ART treatments

**Author, year, [reference #]**	**Country**	**Study design**	**COH**	**Sample size**	**Biological sample(s) used for 25(OH)D assessment**	**Main Outcomes**	**Statistical analysis used for cycle outcome assessment**	**Main finding(s)**	**Main confounding factors considered in the analysis**
Potashnik *et al.*, 1992 [47]	Israel	Prospective cohort	Long agonist protocol	10	Serum; follicular fluid (from “one large follicle”)	Vitamin D and metabolites’ levels throughout COH	Paired *t*-test	1,25(OH)_2_D increased at the end of stimulation. No changes observed for 25(OH)D, 24,25(OH)_2_D	None
Ozkan *et al.*, 2010 [38]	USA	Prospective cohort	Long agonist protocol	84	Serum; follicular fluid (pooled from follicles ≥14 mm)	CPR	Multivariate logistic regression	FF levels of 25(OH)D act as positive independent predictor of CPR	Age
									Race
									BMI
									Embryos transferred (n)
Anifandis *et al.*, 2010 [39]	Greece	Retrospective cohort	Short agonist protocol	101	Serum; follicular fluid (pooled from all follicles)	Glucose levels in follicular fluid; embryo quality; CPR	X^2^-test	Higher FF levels of 25(OH)D are associated with lower FF glucose levels and with lower CPR	Age
									BMI
									Oocytes retrieved (n)
Aleyasin *et al.*, 2011 [40]	Iran	Prospective cohort	Long agonist protocol	82	Serum; follicular fluid (pooled from follicles ≥14 mm after oocyte isolation)	CPR	Multivariate logistic regression	No significant association between FF or serum levels of 25(OH)D and CPR	Variables showing p value ≤ 0.2 on univariate logistic regression
Rudick *et al.*, 2012 [41]	USA	Retrospective cohort	Long agonist; antagonist; microdose flare protocol	188	Serum	CPR	Multivariate logistic regression	Opposite relation between 25(OH)D levels and IVF outcomes by race: higher levels of 25(OH)D associated with higher CPR in non-Hispanic whites and with lower CPR in Asians	Age
									BMI
									Embryos transferred (n, quality)
									Poor ovarian reserve
Firouzabadi *et al.*, 2014 [34]	Iran	Prospective cohort	Long agonist protocol	221	Serum; follicular fluid	Chemical pregnancy rate	Kruskal-Wallis H test	No significant association between FF or serum levels of 25(OH)D and CPR	None
Rudick *et al.*, 2014 [42]	USA	Retrospective cohort	Egg donation recipients	99	Serum	CPR, LBR	Multivariate logistic regression	Higher 25(OH)D levels associated with higher CPR and LBR	Recipient age
									Recipient BMI
									Embryos transferred (n, quality)

### Prevalence of vitamin D deficiency among women undergoing ART procedures

Six cohort studies have characterized serum 25(OH)D status in women undergoing ART procedures and addressed the prevalence of vitamin D deficiency among these patients [34,38-42] (Table 2). In five studies [38-42], serum 25(OH)D was categorised according to clinically accepted ranges for vitamin D deficiency (<20 ng/ml), insufficiency (20–30 ng/ml) and replete (>30 ng/ml) [43]. The observed prevalence of vitamin D deficiency as defined by serum 25(OH)D levels <20 ng/m was 21% [41], 26% [42], 27% [38], 31% [39] and 99% [40] respectively in the five studies. In the study by Firouzabadi et al., vitamin D deficiency was instead exclusively defined as 25(OH)D levels < 10 ng/ml [34], and the prevalence of vitamin D deficiency was 75% with only 7% of patients having replete vitamin D levels (**≥**30 ng/ml) [34]. Interestingly, the studies reporting the highest frequency in vitamin D deficiency (75% for Firouzabadi et al., [34] and 99% for Aleyasin et al., [40]) were both conducted on Iranian women, confirming a strong influence of the socio-economic factors on vitamin D status [44]. After excluding these two studies, the overall mean prevalence of vitamin D deficiency among the population of women who are candidates for IVF procedures was 25% (21-31%), which is slightly lower than that reported for the general population of childbearing age women in the USA (36%) [45]. This observation seems consistent with the demographics of the IVF population, which tends to have higher socio-economic status and education level, which are both factors related to higher vitamin D status [46]. Conversely, the mean observed prevalence of women with replete vitamin D status [serum 25(OH)D levels > 30 ng/mL] was 34% (range 21-42% across studies) after excluding the studies by Firouzabadi et al. [34] and Aleyasin et al. [40].

### Vitamin D status and women’s characteristics

Four studies have evaluated baseline characteristics of patients found to be significantly associated with vitamin D status, but these studies reported conflicting results [38-41]. According to Anifandis et al., populations were homogenous with regards to various patients’ characteristics after grouping for vitamin D status [39]. Conversely, Rudick et al. reported significant differences in age and BMI between women with and without vitamin D deficiency, with patients affected being younger and with higher BMI [41]. In addition, Ozkan et al. also noted an inverse correlation between FF 25(OH)D and BMI (r = −0.25, p = 0.03) [38], while Aleyasin et al. described a linear correlation of serum 25(OH)D levels with age (r = 0.28, p = 0.01) but not with BMI [40]. Three studies have also investigated the influence of ethnicity on 25(OH)D levels [38,41,42]. Of the two studies conducted on conventional IVF populations, one reported Hispanic whites to have significantly lower serum 25(OH)D levels compared to Asians and non-Hispanic whites (p = 0.01) [41] while the other observed, as expected, significantly lower serum 25(OH)D levels among black versus non-black ethnicities (p = 0.001) [38]. The study conducted on recipient women undergoing an oocyte donation program reported lower 25(OH)D levels among Asians and African-Americans compared to Hispanic or non-Hispanic white (p = 0.02) [42].

### Serum and follicular fluid levels of 25(OH)D

Only one small study conducted sequential measurements of serum vitamin D throughout COH for IVF in order to identify cycle-related variations [47], and showed that 1,25-dihydroxyvitamin D concentrations - but not 25(OH)D and 24,25-dihydroxyvitamin D - progressively increased throughout COH [47]. Five studies instead analysed the relationship between serum and follicular fluid (FF) levels of 25(OH)D [34,38-40,47], all confirming that a strong positive correlation exists between peripheral and follicular vitamin D stores (r = 0.74, p < 0.01 according to Potashnik et al., 1992; r =0.94; p < 0.001 according to Ozkan et al.; r = 0.79, p < 0.001 according to Anifandis et al.; r = 0.83, p = 0.001 according to Firouzabadi et al.; r = 0.77, p < 0.001 according to Aleyasin et al.). Of note, this observation seems to suggest that peripheral vitamin D status is a reliable indicator for 25(OH)D availability within the ovary.

### Vitamin D status in women and IVF outcomes: systematic review and meta-analysis

The main characteristics and findings of the six studies that have addressed the role of vitamin D status on IVF/ICSI outcomes [34,38-42] are shown in Table 2. Two studies exclusively investigated the association between FF 25(OH)D levels and ART outcomes. Ozkan et al. were the first reporting a positive association between FF 25(OH)D levels and IVF outcomes, with patients in the highest tertile of FF 25(OH)D distribution being almost four fold more likely to achieve a clinical pregnancy (CP) compared with patients in the lowest tertile after controlling for potentially confounding factors [odds ratio (OR) for CP 3.83; 95% CI 1.20 – 12.28, p = 0.02]. In their study, FF 25(OH)D levels as an independent predictor of CP were also confirmed by means of multivariate logistic regression (confounders-adjusted OR 1.07; 95% CI 1.01 – 1.13, p = 0.01). In marked contrast with these results, Anifandis et al. observed a negative effect of increasing FF vitamin D levels on IVF outcomes. Women with higher (>30 ng/ml) FF 25(OH)D levels showed decreased embryo quality (mean score of embryo quality 5.6 ± 3.6 vs 7.02 ± 2.5 and 7.96 ± 2.6 respectively, p < 0.05) and reduced CPR (14.3% vs 32.7% and 32.3% respectively, p < 0.05) compared to patients with intermediate (20.1-30 ng/ml) or low (**≤**20 ng/ml) FF 25(OH)D levels. Conflicting observations have also been derived from the study by Rudick et al. (2012), in which opposite trends of association were observed according to patients’ ethnicity. In fact, after dividing the population into vitamin D deficient (<20 ng/ml), insufficient (20–30 ng/ml) and replete (>30 ng/ml) depending on serum 25(OH)D levels, vitamin D status was positively associated with increasing CPR in non-Hispanic and Hispanic whites (CPR 21%, 36% and 55% in the deficient, insufficient and replete groups respectively, p = 0.01, in non-Hispanic whites; CPR increasing from 15%, 38% and 68% in the deficient, insufficient and replete groups respectively, p = 0.03, in Hispanic whites). In contrast, an opposite inverse relationship between vitamin D status and IVF success was observed in the Asian ethnicity (CPR 64%, 34% and 14% in the deficient, insufficient and replete groups respectively, p = 0.01). Interestingly, the opposite influence of vitamin D status on IVF outcome was also confirmed on LBR among non-Hispanic whites (increasing from 14% in the deficient group to 27% and 47% in the insufficient and replete groups, respectively, p = 0.01) and Asians (with LBR decreasing from 53% to 25% and 9% across the vitamin D deficient, insufficient and replete groups respectively, p = 0.02), but not among Hispanic whites (p = 0.19) [41]. In a second study, the same authors examined serum vitamin D levels among n = 99 recipients of oocyte donation. After dividing patients into vitamin D deficient (<20 ng/ml), insufficient (20–30 ng/ml) and replete (>30 ng/ml) depending on serum 25(OH)D levels, the authors observed a significant increase in both CPR (37%, 37% and 78% respectively, p = 0.004) and LBR (31%, 30% and 59% respectively, p = 0.04) across the three groups, suggesting a specific effect of 25(OH)D levels on IVF outcomes to be mediated by endometrial receptivity [42]. However, a recent study by Firouzabadi et al., showed increased fertilization rates across groups based on serum vitamin D status. In detail, the fertilization rates associated with serum vitamin D deficiency (<10 ng/ml), insufficiency (10–29 ng/ml) and sufficiency (30–100 ng/ml) were 43%, 53%, and 59%, respectively (p = 0.05). Of note, no statistical significance was reached and vitamin D status was in general low in the population studied - with only 7% of women being vitamin D sufficient [34]. Similarly, results from the study by Aleyasin et al., which showed that CPR did not change significantly across groups based on increasing tertiles of FF vitamin D levels (29%, 30% and 30% respectively, p = 0.9) are difficult to interpret because all women in the population except for one (n = 81/82) were vitamin D deficient in the serum [40].

**Figure 2:**
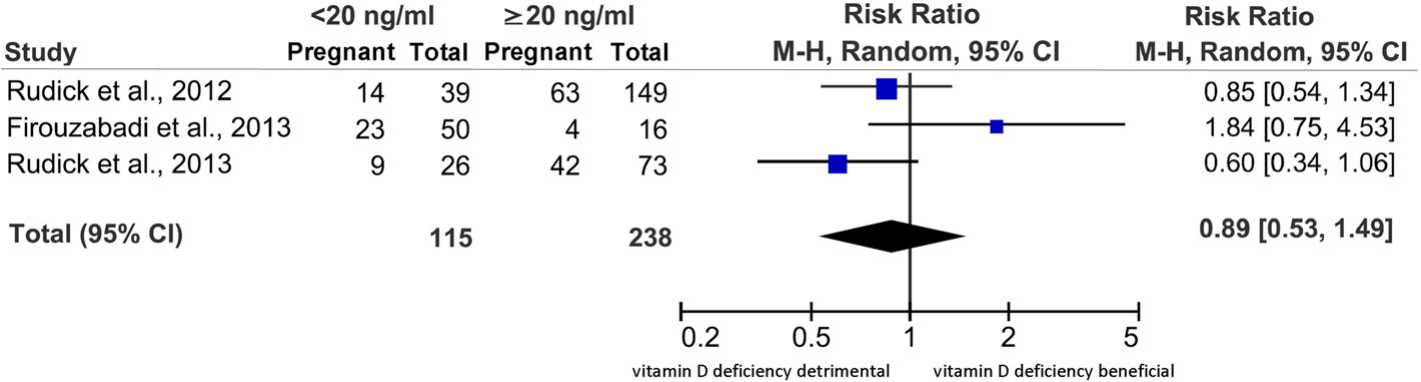
**Forest plot of meta-analysis results.** Included studies account for n = 353 women. A clinical pregnancy was achieved in 46/115 (40%) women with vitamin D deficiency, and in 109/238 (46%) in those with sufficient serum 25(OH)D levels, yielding a RR of 0.89 (95% CI 0.53 to 1.49) showing a lower but not statistically significant likelihood of CP for vitamin D deficient women undergoing IVF/ICSI procedures compared with vitamin D sufficient patients. Please note that in the study by Firouzabadi et al. all patients with vitamin D deficiency had serum 25(OH)D levels < 10 ng/ml while all those with sufficient serum 25(OH)D levels had serum 25(OH)D levels > 30 ng/ml. CI = confidence interval. M-H = Mantel-Haenszel. RR = Risk Ratio.

When pooling data for meta-analysis, data on the effect of vitamin D serum deficiency (<20 ng/ml) on CPR after IVF/ICSI procedures were extracted [34,40-42]. Categorisation of patients was not possible in the study by Aleyasin et al. due to the fact that all patients except for one were deficient [40]. Overall, the remaining three studies account for n = 353 women undergoing IVF/ICSI procedures and fresh embryo transfer, of which n = 115 were vitamin D deficient [as defined by serum 25(OH)D levels <20 ng/ml] and n = 238 were vitamin D sufficient [serum 25(OH)D levels **≥**20 ng/ml]. Results are shown in Figure 2. A clinical pregnancy was achieved in 46/115 (40%) women with vitamin D deficiency, and in 109/238 (46%) in those with sufficient serum 25(OH)D levels. The RR of clinical pregnancy ranged from 0.60 to 1.84 [48,49] across studies, and pooling of the results yielded a common RR of 0.89 (95% CI 0.53 to 1.49), showing a lower but not statistically significant likelihood of CP for vitamin D deficient women undergoing IVF/ICSI procedures compared with vitamin D sufficient patients (Figure 2).

#### Vitamin D status in men and ART outcomes

No data are currently available regarding vitamin D and semen performance in regards to clinical outcomes including fertilization rate, embryo quality, pregnancy rate and LBR in the context of IVF treatments. Further studies conducted on spermatozoa from infertile men are utterly needed to allow the identification of a potential role of vitamin D in influencing treatment outcomes for couples undergoing IVF for male factor infertility. Of note, based on the available data in infertile men in general, there is an ongoing randomized controlled trial (RCT) by Blomberg Jensen’s group (ClinicalTrials. gov Identifier: NCT01304927) testing the benefits of vitamin D supplementation on semen parameters.

### Comments

The issue of whether vitamin D levels are reliable predictors of ART outcomes is still controversial. Evidence is still poor, because no RCTs are currently available and results of existing small cohort studies are very heterogeneous. Consequently, the meta-analysis performed herein fails to identify a significant association between vitamin D status and CPR after ART. Marked differences in exposure categorisation, analytic approaches, outcomes considered and general methodological design characterize the identified studies. More specifically:

1. Main outcomes varied greatly among the various studies including both biological and clinical outcomes such as embryo parameters and pregnancy rates.

2. A matter of concern may be the threshold used to define insufficiency. The commonly accepted cut-off value for vitamin D deficiency [serum 25(OH)D < 20 ng/ml] has been established based on bone health in the general population, and may not be appropriate to the peri-conceptional period, with some authors proposing that circulating levels of 25(OH)D should be as high as 40–60 ng/ml during gestation [48]. Therefore, a higher availability of vitamin D as compared to the standard cut-off value of 20 ng/ml might also be required in the population of infertile couples undergoing IVF, and further analysis of the critical threshold for serum 25(OH)D levels with regards to the peri-conceptional period is needed.

3. Some clinical conditions that are commonly encountered among IVF candidates (including younger or older age, higher BMI, PCOS, endometriosis, and uterine leiomyomas) have been related to significantly altered serum 25(OH)D levels. These conditions are also associated with relevant differences in prognosis with regards to IVF outcomes. While the potential confounders represented by age and BMI can be mostly excluded because all studies properly controlled for both covariates in their statistical analyses (with the only exception of Firouzabadi et al. who did not report any data on patients’ BMI), most of the available studies do not give sufficient information to exclude potential biases caused by the different prevalence of the other factors (e.g. PCOS, endometriosis, and uterine leiomyomas).

4. Not all the currently available studies have controlled for potential confounders affecting implantation and pregnancy rates such as the number and quality of embryos transferred.

5. Variations exist in the assays used for 25(OH)D assessment among studies as demonstrated by measurement variability and large discrepancies in concentrations observed in different laboratories [49].

6. Finally, relevant factors such as duration of vitamin D deficiency or source of vitamin D (previous supplementation, sun exposure and diet) [43] have never been investigated in any of the studies.

Due to the presence of several sources of controversy in the interpretation of the relationship between vitamin D deficiency and poor IVF outcome, we have employed the nine criteria proposed by Austin Bradford Hill, which still stand as foundation milestones for addressing causation conditions [50]. Five (“specificity“, “temporality“, “biological plausibility“, “experimental evidence“, “analogy“) out of nine Hill’s criteria for causality are indeed fulfilled, two (“consistency” and “biological gradient”) are fulfilled by all currently available studies except for one, while the “specificity” criterion cannot be determined due to insufficient data and the “strength of association” criterion is not fulfilled likely due to the small sample size of the studies and the low magnitude of the effect. Hence, according to the Hill’s criteria, a causal relationship between vitamin D deficiency and negative outcomes should be recognised, but further research is needed to determine the strength of the association and the specific underlying mechanisms [50].

In summary, consistent heterogeneity exists among available studies and important factors need to be considered in this regard such as insufficient sample size, ethnicity, BMI, countries of origin, socio-economic status and the presumably mild magnitude of the effects. This latter aspect should however not disregard the potential relevance of vitamin D status in an IVF setting for two main reasons. Firstly, there is growing evidence that maternal vitamin D status may influence pregnancy outcome, with consistent evidence demonstrating a role of vitamin D insufficiency in pre-eclampsia and preterm birth [5]. Albeit less robust, there is also some data suggesting a relationship with gestational diabetes mellitus and late growth restriction [5]. Hence, the relevance of an advantage of vitamin D sufficiency in increasing the chances of pregnancy in IVF cycles would be strengthened by the potential, subsequent advantage in terms of improvements in obstetrics complications and neonatal health even if the magnitude of the effect is still to be measured [51]. Secondly, vitamin D deficiency or insufficiency is easily amenable to correction. Oral vitamin D supplementation at therapeutic doses is indeed a simple and cheap intervention without significant side effects. Importantly, recent studies have shown that maternal supplementation with vitamin D during second and third-trimester pregnancy at doses up to 4000 IU is safe and can effectively improve maternal vitamin D status [52]. However, results of the ongoing RCTs on vitamin D supplementation prior to IVF procedures are not yet available.

## Conclusions

Our meta-analysis suggests that vitamin D status assessment and supplementation prior to IVF is currently not recommended. Further studies on this topic are required. In fact, despite a trend for a negative effect of vitamin D deficiency on ART outcomes, results are still controversial and large cohort studies properly adjusting for confounders are required to assess the influence of vitamin D deficiency and to properly estimate the magnitude of the effect. If confirmed, RCTs will be necessary to establish future indications for routine assessment of vitamin D status and supplementation prior to ART. Interestingly, Lindqvist and colleagues are currently testing the effects of high (1000 IU) and low (500 IU) dose vitamin D supplementation on 1000 women initiating IVF treatment in Sweden in the registered prospective double-blind randomised trial entitled “Vitamin D during IVF” [ClinicalTrials.gov: NCT01019785], but results are still not available. On the other hand, there is the need for independent trials in different populations as the benefits of supplementation may markedly differ among populations.

## Abbreviations

ART: Assisted reproductive technology; BMI: Body mass index; COH: Controlled ovarian hyperstimulation; CP: Clinical pregnancy; CPR: Clinical pregnancy rate; FF: Follicular fluid; ICSI: Intra-cytoplasmic sperm injection; IVF: In vitro fertilization; LBR: Live birth rate; OR: Odds ratio; RR: Risk ratio; VDR: Vitamin D receptor.

## Competing interests

The authors declare that they have no competing interests.

## Authors’ contributions

The review was conceived by PV and MC. Data were collected, analyzed and interpreted by VSV, PV, EP and LP, who also drafted the article. ES, AP and MC made substantial contributions to further interpretation and discussion of data and to article revision. All authors read and approved the final manuscript.
